# Beneath the Bark and Beyond the Known: The First Record of *Tineobius* Ashmead (Hymenoptera: Chalcidoidea: Eupelmidae) in China with a Description of Two New Species

**DOI:** 10.3390/insects16060597

**Published:** 2025-06-05

**Authors:** Zixuan Li, Haoran Liao, Shirui Xu, Haitian Song, Lingfei Peng

**Affiliations:** 1 Biological Control Research Institute, Fujian Agriculture and Forestry University, Fuzhou 350002, China; lizixuan1813@163.com (Z.L.); liaohaoran76@gmail.com (H.L.); xushirui10050@163.com (S.X.); 2 China Fruit Fly Research and Control Center of FAO/IAEA, Fuzhou 350002, China; 3 State Key Laboratory of Ecological Pest Control for Fujian and Taiwan Crops, Fuzhou 350002, China; 4 Fujian Academy of Forestry, Fuzhou 350012, China; haitiansong@126.com

**Keywords:** Asia, ASAP, COI, eupelmids, integrated taxonomy, key, male

## Abstract

The genus *Tineobius* Ashmead (Hymenoptera: Eupelmidae) is recorded for the first time in China based on two new species, *Tineobius* (*Tineobius*) *elpisios* Li & Peng sp. nov. and *Tineobius* (*Tineobius*) *victor* Li & Peng sp. nov., along with two newly recorded species, *Tineobius (Tineobius) brachartonae* (Gahan, 1927) and *Tineobius (Tineobius) longicauda* (Ferrière, 1938). Descriptions and illustrations are provided for all female specimens of the four species. Additionally, the males of *T. victor* sp. nov. and *T. brachartonae* are described and illustrated in detail, and a key to Asian species based on females is provided.

## Simple Summary

The genus *Tineobius* Ashmead, 1896 (Hymenoptera: Eupelmidae) is reported for the first time in China, with the discovery of two new species, *Tineobius (Tineobius) elpisios* Li & Peng sp. nov. and *Tineobius (Tineobius) victor* Li & Peng sp. nov., and two newly recorded species, *Tineobius (Tineobius) brachartonae* (Gahan, 1927) and *Tineobius (Tineobius) longicauda* (Ferrière, 1938). Detailed morphological descriptions and illustrations are provided for females of all four species, along with the first detailed accounts of males for *T. victor* and *T. brachartonae*. This study employs an integrative taxonomic approach, combining morphological data with COI DNA barcoding, to address historical challenges in sexual association and species delimitation within the genus. A diagnostic key to Asian *Tineobius* species is also presented, enhancing identification tools for this parasitoid group. These findings significantly expand the known distribution of *Tineobius* in Asia and underscore the value of molecular methods in resolving taxonomic uncertainties in sexually dimorphic Eupelmidae.

## 1. Introduction

*Tineobius* was established by Ashmead in 1896 for his newly described species *T. citri* from Queensland, Australia. Nowadays, *Tineobius* is distributed across the Afrotropical, Oriental, Australasian, and Palaearctic regions with 31 known species [1,2]. Historically, species of *Tineobius* were often described under other genera, such as *Metapelma* Westwood, 1835. Today, *Metapelma* has been classified under Metapelmatidae [3] with some species being transferred to *Tineobius* by Bouček [4], Fusu, and Ribes [2].

Bouček [4], Gibson [1], Fusu, and Ribes [2] have made significant contributions to the understanding of *Tineobius*. Bouček [4] synonymized *Australeupelmus* Girault, 1921 and *Anastatoidea* Gahan, 1927 under *Tineobius*. Then, Gibson [1] conducted a comprehensive review of the world genera of Eupelmidae, and divided *Tineobius* into three subgenera: *Duanellus* Bouček, 1988; *Tineobius* Ashmead, 1896; and *Progenitobius* Gibson, 1995. Recently, Fusu and Ribes [2] transferred nine species into *Tineobius*, assigned subgenera to existing species, and excluded certain species from *Tineobius*, and subsequently reassigned them to other genera. Additionally, they compiled a checklist of all described species of *Tineobius* worldwide. Currently, the genus *Tineobius* comprises 31 species, with the subgenus *Tineobius* containing 27 species, while *Progenitobius* and *Duanellus* respectively have two species.

The taxonomy of Eupelmidae is challenging due to the sexual dimorphism in these parasitoids. Males of Eupelminae often exhibit morphological similarities to certain Pteromalidae species, resulting in many species being described based on a single sex because establishing the sexual association within the same species is difficult. In the past, the sexual association of Eupelmidae species has primarily relied on rearing them from their host [5,6,7], or just based on morphological identification [8].

The situation is also true for *Tineobius*. For instance, both sexes of *Tineobius capensis* (Ferrière, 1938) [9] were collected from the cocoons of *Latoia albifrons* Guérin-Méneville, 1844 (Lepidoptera: Limacodidae). Similarly, both sexes of *Tineobius mandrakae* (Risbec, 1952) [10] were obtained from the galls of *Albizzia* sp., and both sexes of *Tineobius seyrigi* (Ferrière, 1936) were obtained from the cocoon of *Borocera* sp. (Lepidoptera: Lasiocampidae), which were parasitized by *Enicospilus cohacarus* Gauld & Mitchell, 1978 (Hymenoptera: Ichneumonidae). However, the male and female associations of *Tineobius beharae* (Risbec, 1952) [10] were not produced by the same host rearing. Furthermore, in the original description of *Tineobius albopalpalis* (Brues, 1907), the sex of the holotype was incorrectly identified as female (Fusu and Ribes 2017). However, these taxonomic challenges can be addressed by employing DNA barcoding techniques. In fact, molecular data have been used in the fields of taxonomy, phylogenetics, and the confirmation of sexual and host associations in Hymenoptera and other groups [11,12,13].

In this study, based on an integrative taxonomic approach that combines molecular and morphological data, the genus *Tineobius* is reported for the first time with four species from China, including two new species and two newly recorded species. In addition to the descriptions of all females, the males of *T. victor* sp. nov. and *T. brachartonae* are also described and illustrated in detail. A key to the Asian *Tineobius* species is also provided.

## 2. Materials and Methods

### 2.1. Specimens

In this study, twenty-five specimens were collected from 2015 to 2025 by Malaise trap, Flight interception trap, direct collection, and sweeping in the field, and then stored in 95% ethanol at −20 °C until DNA extraction. Specimens were numbered with a unique DNA voucher as “DNA XXX”.

All specimens were deposited at the Biological Control Research Institute, Fujian Agriculture and Forestry University, Fuzhou, Fujian, China (FAFU). New distribution records presented in this paper are indicated by an asterisk (*).

### 2.2. Imaging

The descriptions were based on specimens that were examined with a Leica M165C stereo microscope (Leica Microsystems AG, Heerbrugg, Switzerland) and a Leica LED 5000 HDI dome light source (Leica Microsystems AG, Heerbrugg, Switzerland) and imaged with a Leica MC170 HD digital camera (Leica Microsystems AG, Heerbrugg, Switzerland) attached to the microscope. The serial images obtained were combined with Zerene Stacker 1.04 software (Zerene Systems, LLC, Richland, Washington, DC, USA). Adobe Photoshop CC2019 (Adobe Systems Incorporated, Los Angeles, CA, USA) was used to edit pictures and enhance their clarity.

### 2.3. Morphological Terminology and Abbreviations

The terminology for structure, sculpture, and color descriptions follows Gibson [7]. The abbreviations used in the description are as follows:

cc = costal cell of the forewing.

LOL = minimal distance between the anterior and the posterior ocellus.

MPOD = maximum diameter of the posterior ocellus.

mv = marginal vein of the forewing.

OOL = minimal distance between the posterior ocellus and the inner orbit.

pmv = postmarginal vein of the forewing.

POL = minimal distance between the posterior ocelli.

stv = stigma vein of the forewing.

Gt_n_ = the serial number of gastral tergite.

### 2.4. Mapping the Distribution

To visualize the geographical distribution of *Tineobius* species across China, we created a distribution map using QGIS (version 3.38.3), an open source Geographic Information System software. All geographic coordinates were converted to the WGS 84 coordinate system to maintain uniformity. The base map was obtained from Tianditu (www.tianditu.gov.cn), the official Chinese online map service, which provides high-resolution satellite imagery and geographic data. The Tianditu map layer was integrated into QGIS using the XY Tiles function, facilitating a comprehensive visualization of the species’ distribution. All the data were derived from the specimens of FAFU.

### 2.5. DNA Extraction, Amplification, and Sequencing

A total of 23 specimens representing 7 morphospecies were used for DNA barcoding analysis (see Table 1). Whenever available, both female and male specimens of a species were included. DNA extraction was carried out using a DNeasy Blood & Tissue Kit (Qiagen, Hilden, Germany), following the manufacturer’s instructions, with some modifications: (i) the specimen was not crushed, but the gaster was pierced with an insect pin to create a hole, preserving the specimen while maximizing the quantity of DNA recovered; (ii) the incubation was prolonged to at least 12 h at 56 °C in a thermo-shaker; and (iii) the adsorption column was equilibrated at room temperature for 3 min before the eluent was added. A cytochrome oxidase subunit I (COI) barcode fragment was amplified using the primers LCO1490 (5′-GGTCAACAAATCATAAAGATATTGG-3′) and HCO2198 (5′-TAAACTTCAGGGTGACCAAAAAATCA-3′) [14].

COI PCRs were performed in a 50 µL reaction volume containing 25 µL of 2*×* Gflex PCR Buffer (including Mg^2+^ and dNTP plus), 1 µL of Tks Gflex DNA Polymerase (Takara Biomedical Technology, Beijing, China), 0.5 µL of each primer (10 µM), 17 µL of ddH2O, and 6 µL of DNA template. The PCR conditions were as follows: initial denaturation at 94 °C for 5 min, followed by 35 cycles of 94 °C for 30 s, 49 °C for 30 s, and 72 °C for 1 min, with a final extension at 72 °C for 10 min. After electrophoresis on 2% agarose gel, the COI PCR products were sent to Tsingke Biotech (Beijing, China) for bidirectional sequencing. Geneious R11 (Auckland, New Zealand) was used to check the quality of the peak pattern, manually correct and assemble the sequencing results, and export them in the FASTA format. All sequences generated from this study were deposited in GenBank (accession numbers, see Table 1).

### 2.6. Sequence Analysis and Molecular Species Delimitation

In this study, a maximum likelihood (ML) tree was constructed using the genus *Metapelma* as an outgroup. The tree was based on twenty-three newly obtained COI genes, one *Tineobius* COI gene downloaded from GenBank, and one *Metapelma* COI gene downloaded from BOLD systems. Sequences were aligned with MAFFT v7.505 using the ‘G-INS-I’ strategy and normal alignment mode [15]. Subsequently, ModelFinder v2.2.0 was used to select the most suitable substitution model using Bayesian Information Criterion (BIC) [16]. The ML tree was constructed using IQ-TREE [17] in the PhyloSuite platform [18,19]. The analysis was conducted under the TIM+G4+F substitution model with 10,000 ultrafast bootstrap replicates [20]. The resulting ML tree was visualized using iTOL v.7 [21]. Nodal support for the ML analysis was assessed by the frequency of clade occurrence across the resampled datasets and is expressed as bootstrap values (BVs) in percentages. BVs > 90% were considered to indicate strong nodal support, while those ranging from 70% to 90% were regarded as moderately good support.

Assemble Species by Automatic Partitioning (ASAP) was employed for the delimitation of molecular species [22]. ASAP efficiently delimits species from large single-locus sequence datasets by calculating pairwise genetic distances, applying hierarchical clustering, and integrating *p*-values with barcode gap widths to compute ASAP scores. This analysis was conducted using the web server available at https://bioinfo.mnhn.fr/abi/public/asap/asapweb.html (accessed on 19 April 2025).

## 3. Results

### 3.1. *COI* Sequence Analysis

**Figure 1:**
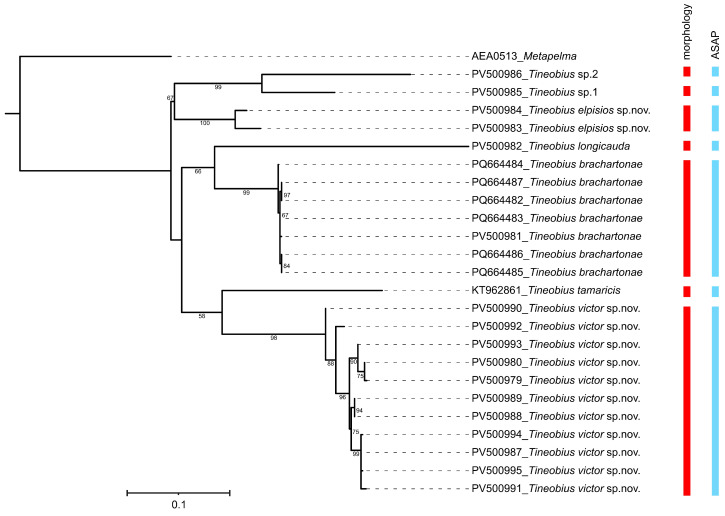
Maximum likelihood tree based on the COI gene and the species delimitation was determined using two methods. Only bootstrap values exceeding 50% are labeled on the tree.

The present study generated 23 COI sequences with an average of 636 bp. Voucher specimens of these 23 sequences were subjected to further morphological examination, and seven species belonging to *Tineobius* were recognized, including two new species described below (Table 1). The K2P distances (Tables S1 and S2) showed a larger intergroup than intragroup distance for the COI sequences. The intraspecific pairwise distances ranged from 0 to 3.2%. The interspecific pairwise distances ranged from 12.3% to 24.2%. The delimitations of all studied species are congruent with the morphological identification results in the ASAP method (Figure 1).

### 3.2. Taxonomy

*Tineobius* Ashmead, 1986.
*Tineobius* Ashmead, 1896: 14–15. Type species: *Tineobius citri* Ashmead. Original designation [23].
*Australeupelmus* Girault, 1921: 3 [24]; Synonymy by Bouček, 1988: 564 [4].
*Anastatoidea* Gahan, 1927: 122 [25]; Synonymy by Bouček, 1988: 564 [4].

**Diagnosis.** See Fusu and Ribes [2].

**Distribution.** Afrotropical (Madagascar, Namibia, South Africa), Oriental (*China, Indonesia, Malaysia, Pakistan, Philippines, Sri Lanka, Thailand), Australasian (Australia, Fiji, Micronesia, Palau, Solomon Islands), and Palaearctic (*China, Spain).

### 3.3. Description of Species

#### 3.3.1. *Tineobius (Tineobius) brachartonae* (Gahan, 1927) n. rec. (Figure 2, Figure 3 and Figure 4)

*Anastatoidea brachartonae* Gahan, 1927: 13–15 [25].
*Tineobius brachartonae*; Bouček, 1988: 564 [4].
*Tineobius (Tineobius) brachartonae*; Fusu & Ribes, 2017: 12 [2].

**Figure 2:**
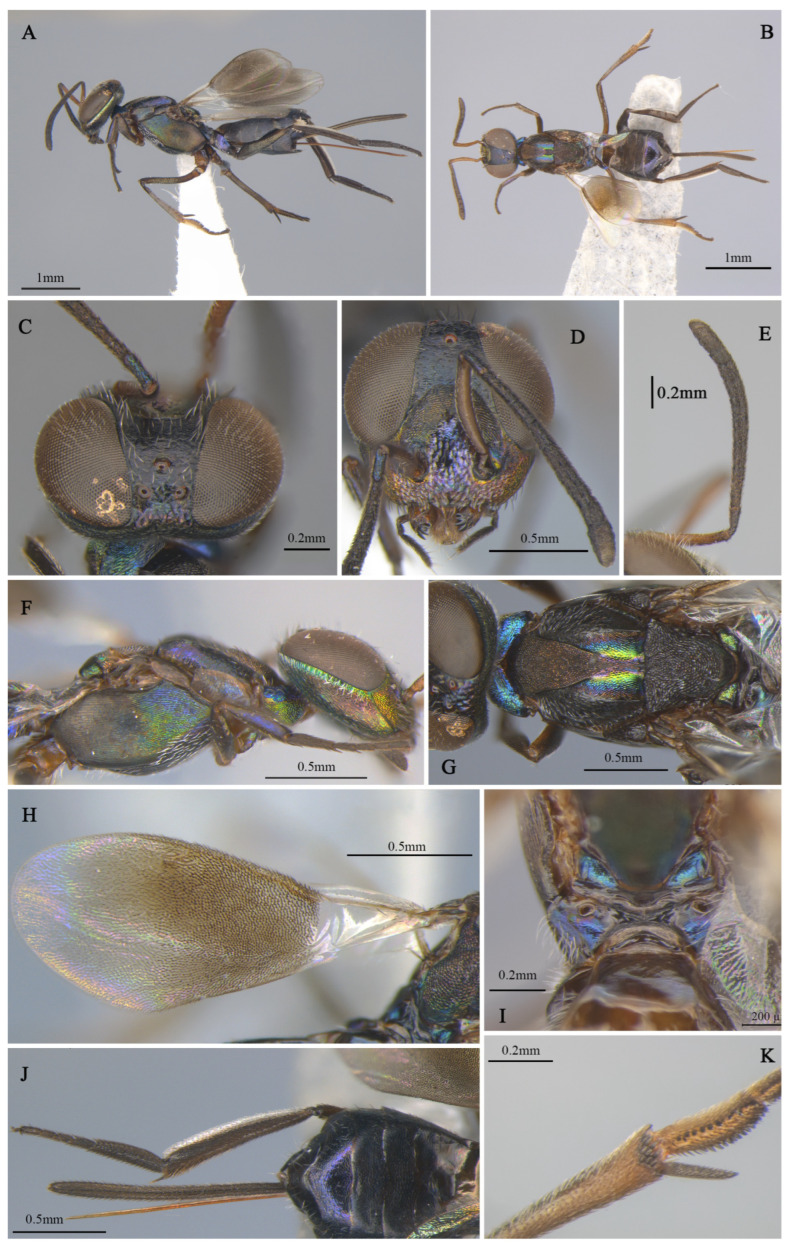
*Tineobius brachartonae* (Gahan, 1927) n. rec.: (**A**) body, lateral; (**B**) body, dorsal, (**C**) head, dorsal; (**D**) head, frontal; (**E**) antenna; (**F**) mesosoma, lateral; (**G**) mesosoma, dorsal; (**H**) forewing; (**I**) propodeum; (**J**) gaster, dorsal; (**K**) apex of mesotibia, ventral ((**A**) from FAFU-DNA1108; (**B**–**H**), (**J**–**K**) from FAFU-DNA1098; (**I**) from FAFU-DNA 481).

**Material examined.** 6♀1♂ (FAFU), Zhangjiangkou Mangrove National Nature Reserve, Yunxiao, Zhangzhou, Fujian province, China|13 January to 17 October 2022|Malaise trap/DNA1081, 1098, 1103, 1106, 1108, 1109 (♀), 1104 (♂); 1♀ (FAFU), Daiyunshan National Nature Reserve, Dehua, Quanzhou, Fujian province, China 5|October 2015|Malaise trap/DNA481.

**Figure 3:**
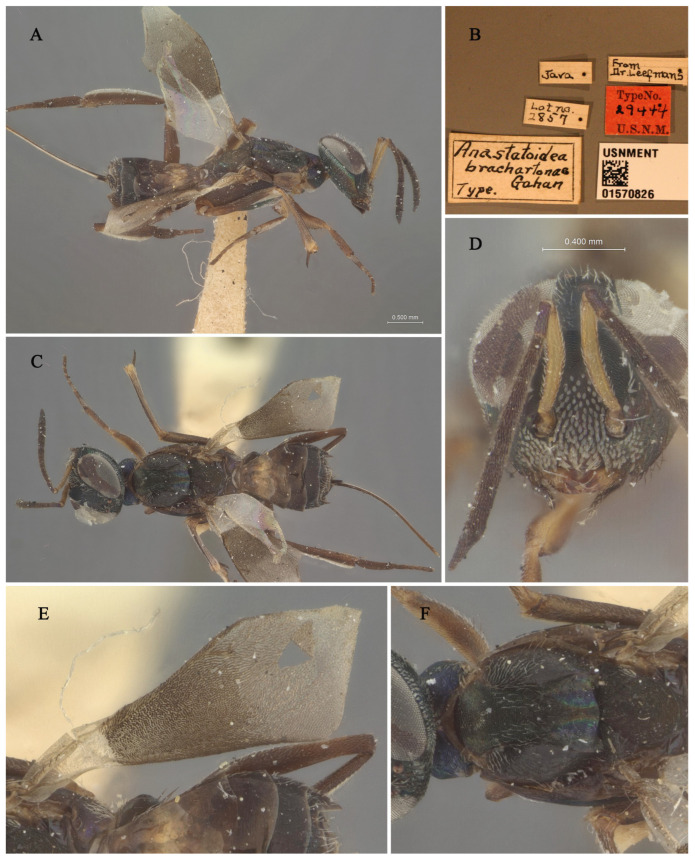
Holotype specimen of *Tineobius brachartonae*: (**A**) body, lateral; (**B**) label; (**C**) body, dorsal; (**D**) head, frontal; (**E**) fore wing; (**F**) mesosoma, dorsal (collection number: USNMENT 01570826; Orrell T, Informatics and Data Science Center—Digital Stewardship (2024). NMNH Extant Specimen Records (USNM, USA). Version 1.93. National Museum of Natural History, Smithsonian Institution. Occurrence dataset https://doi.org/10.15468/hnhrg3 accessed via GBIF.org, accessed on 24 December 2024; https://www.gbif.org/occurrence/1321900501, accessed on 24 December 2024).

**Brief description of female**. Head dark, scrobal depression (Figure 2D), and gena (Figure 2F) with slight greenish-yellow metallic luster, interantennal prominence and purple to reddish-purple lower face, with short, translucent, scalelike setae (Figure 2D). Antenna (Figure 2E) with yellowish-brown scape, pedicel blue-metallic luster, flagellum black. Pronotum black with strong-blue metallic luster. Mesonotum (Figure 2G) dark, except posterior depression region with strong bluish-green and coppery metallic lusters. Acropleuron (Figure 2F) bare, anterior region with alutaceous sculpture and posterior mesh-like longitudinally alutaceous coriaceous sculpture. Mesopectus with intensely white hair-like setae. Propodeum (Figure 2I) slightly emarginate anteromedially (cf. Gibson 1995, Figure 201) to anterior margin straight (cf. Gibson 1995, Figure 202) [1], plical region transverse almost without depression. Legs mostly dark-brown to black, anterior and median coxa mostly testaceous, hind coxa purple. Mesotibia with 10–13 apical pegs (Figure 2K) in 2 or 3 rows. Basal cell of forewing bare (Figure 2H), setae below marginal and stigma vein dense-brown and scalelike, beyond apex of stigma vein hair-like. Gaster (Figure 2J) dark, ovipositor sheaths dark, almost equal in length to gaster.

**Figure 4:**
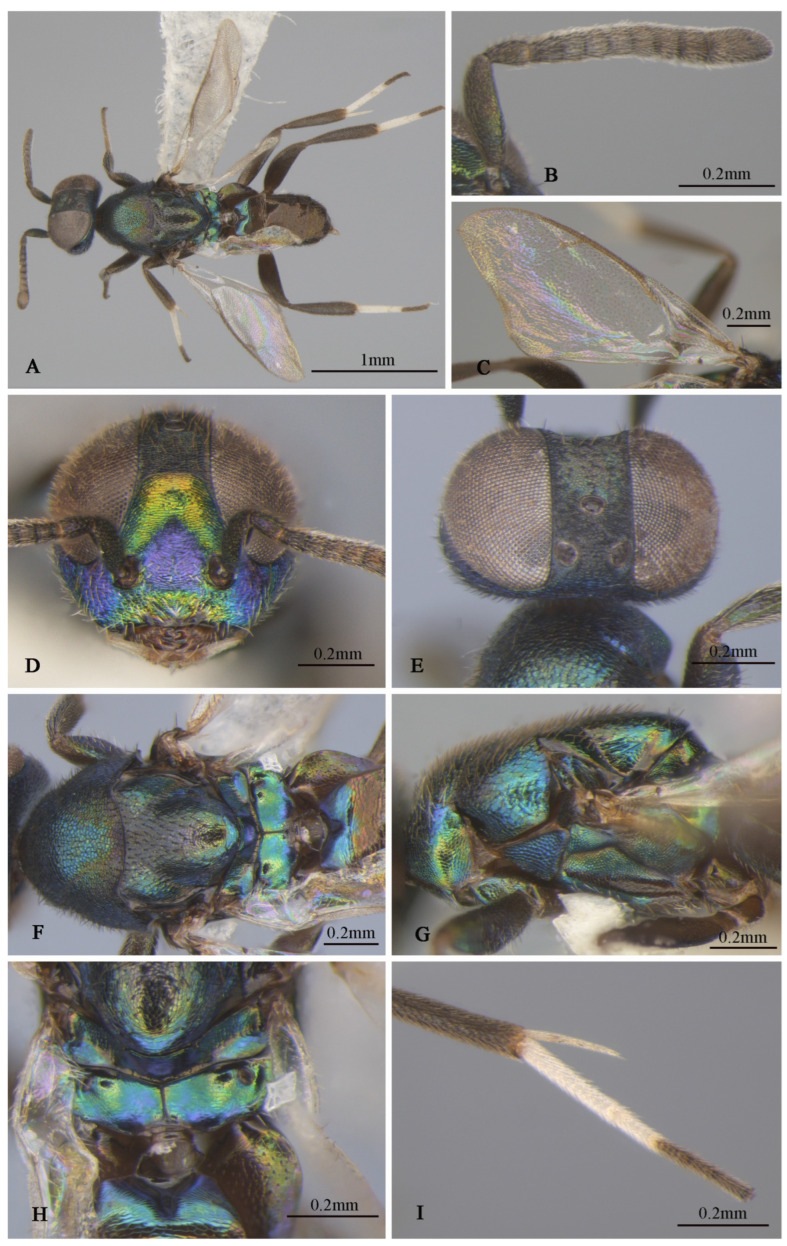
*Tineobius brachartonae* (Gahan, 1927) n. rec. (FAFU-DNA1104): (**A**) body, dorsal; (**B**) antenna; (**C**) forewing; (**D**) head, frontal; (**E**) head, dorsal; (**F**) mesosoma, dorsal; (**G**) mesosoma, lateral; (**H**) propodeum; (**I**) apex of mesotibia, dorsal.

**Description of male**. Body length about 2.3 mm. Head (Figure 4D,E) with green-and-blue metallic lusters, gena and interantennal prominence with purplish-blue metallic luster, scrobal depression with green to greenish-yellow metallic luster, green lower face with greenish-yellow metallic lusters, dark-brown maxillary palpi, apical paler, frons and vertex dark with slightly metallic luster. Eyes and frons (Figure 4E) densely setose with brown hair-like setae, gena and lower face (Figure 4D) densely setose with pale-gray hair-like setae. Frons coriaceous, scrobal depression and interantennal prominence reticulate–imbricate transversely, lower face and gena imbricate. In frontal view, wider head 1.1× height, distance between eyes below 2.6× distance between eyes above, distance between toruli 2.0× distance between torulus and clypeal edge, and 3.3× distance between torulus and eye. In dorsal view, head wider 1.6× length, interocular distance 0.3× head width. In lateral view, malar space 0.3× height of eye. OOL: POL: LOL: MPOD = 1.0: 7.1: 7.4: 6.0. Scrobal depression ^-shaped with dorsal margin wide. Antenna (Figure 4B) dark in color, scape and pedicel with slightly greenish-yellow metallic luster; relative length (width) of scape: 56.8 (18.3), pedicel: 20.5 (12.7), 1st to 8th flagellomeres: 5.4 (10.0), 10.7 (12.2), 12.0 (13.2), 10.7 (13.9), 12.2 (13.7), 12.0 (14.4), 12.0 (14.4), 14.1 (16.3), and clava 32.7 (17.6).

Mesosoma (Figure 4F,G) generally with greenish-blue metallic luster, notaulus conspicuous, mesoscutal medial-lobe region triangular with greenish-blue metallic luster, dark mesoscutal lateral lobe ranging from greenish-yellow to purple metallic lusters, dark-purple scutellum with greenish-blue metallic luster, acropleuron, mesepisternum, mesepimeron, and metapleuron bare, with variable greenish-blue to greenish-yellow metallic lusters from different angles. Axilla and scutellum non-separated, scutellum convex significantly with evenly distributed dark hair-like setae at the front end, lateral sides of pronotum and mesoscutum (Figure 4F) with densely brown setae; prepectus (Figure 4G) reticulate–imbricate with blue metallic luster. Pronotum and mesoscutum reticulate, mesepisternum, acropleuron, mesepimeron and metapleuron reticulate–imbricate. Propodeum (Figure 4H) with variable greenish-blue metallic luster, coriaceous sculptured, several long white setae around spiracle. Forewing (Figure 4C) transparent, light-brown stigma vein, lighter than marginal vein, evenly setose, basal cell with three rows of setae along mediocubital fold, relative length of cc: mv: pmv: stv = 3.7: 2.4: 1.4: 1.0. Legs (Figure 4A) dark except mesotarsus (Figure 4I) and metatarsus pale gray to dark brown, first and second segments pale gray, third, fourth, and fifth segments dark brown, mesotibia spur pale gray.

Gaster dark with variable coppery metallic luster from different angles, except Gt_1_ with distinct blue metallic luster.

**Distribution.** *China (Fujian), Malaysia, Sri Lanka, Indonesia.

Observation record: China (Taiwan).

**Biology.** According to the original description, this species is a primary parasitoid of the larvae or pupae of *Brachartona catoxantha* Hampson, 1893 (Lepidoptera: Zygaenidae) in Indonesia. When functioning as a secondary parasitoid, it parasitizes *Degeeria albiceps* Macquart, 1851 (Diptera: Tachinidae), *Bessa remota* (Aldrich, 1925) (Diptera: Tachinidae), *Apanteles* sp. (Hymenoptera: Braconidae), and an Ichneumonidae species. The host of this species in China is unknown.

**Remarks.** We examined the type specimens through publicly accessible data hosted on the Global Biodiversity Information Facility (GBIF). The forewing of the type specimens has a special-shaped setae (Figure 3E), and also white bristles of a similar shape on the face (Figure 3D). Gahan [25] described these setae as scalelike and mentioned that a similar type of setae is also present in *Tineobius superbus* (Dodd, 1917), which is from Townsville, Australia. Upon a careful comparison of Gibson’s description of the head (cf. pp. 284, 288, 291, 327, Figures 41 and 42) [1] and Fusu and Ribes’s description [2] of the wings (cf. pp. 8–9), we interpret the term “scalelike” as having the same meaning as “spatulate”. Thus, the known species of *Tineobius* in Asia that also have scalelike (spatulate) setae on their forewings include *Tineobius philippinensis* (Ferrière, 1938) and *Tineobius indicus* (Ferrière, 1938).

Gahan provided a detailed description of the species based on four female specimens. In this study, we identified a male of this species based on COI analysis.

#### 3.3.2. *Tineobius (Tineobius) elpisios* Li & Peng sp. nov. (Figure 5 and Figure 6)

Zoobank: urn:lsid:zoobank.org:act:3B76193A-EAFC-4A89-BDE6-03296ECC6696.

**Figure 5:**
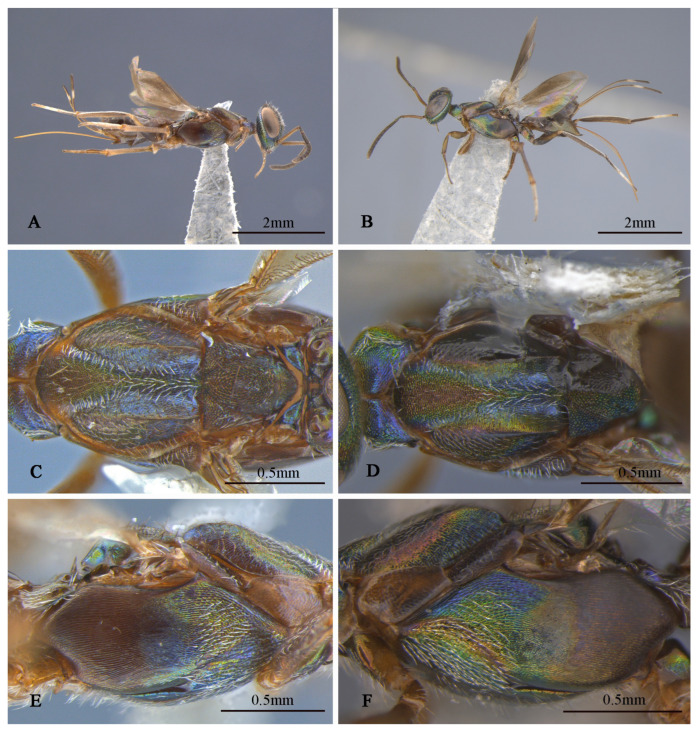
*Tineobius (Tineobius) elpisios* Li & Peng sp. nov.: (**A**) body, lateral; (**B**) body, lateral; (**C**) mesosoma, dorsal; (**D**) mesosoma, dorsal; (**E**) mesosoma, lateral; (**F**) mesosoma, lateral ((**A**,**C**,**E**) from FAFU-DNA962, holotype; (**B**,**D**,**F**) from FAFU-DNA1062, paratype).

**Figure 6:**
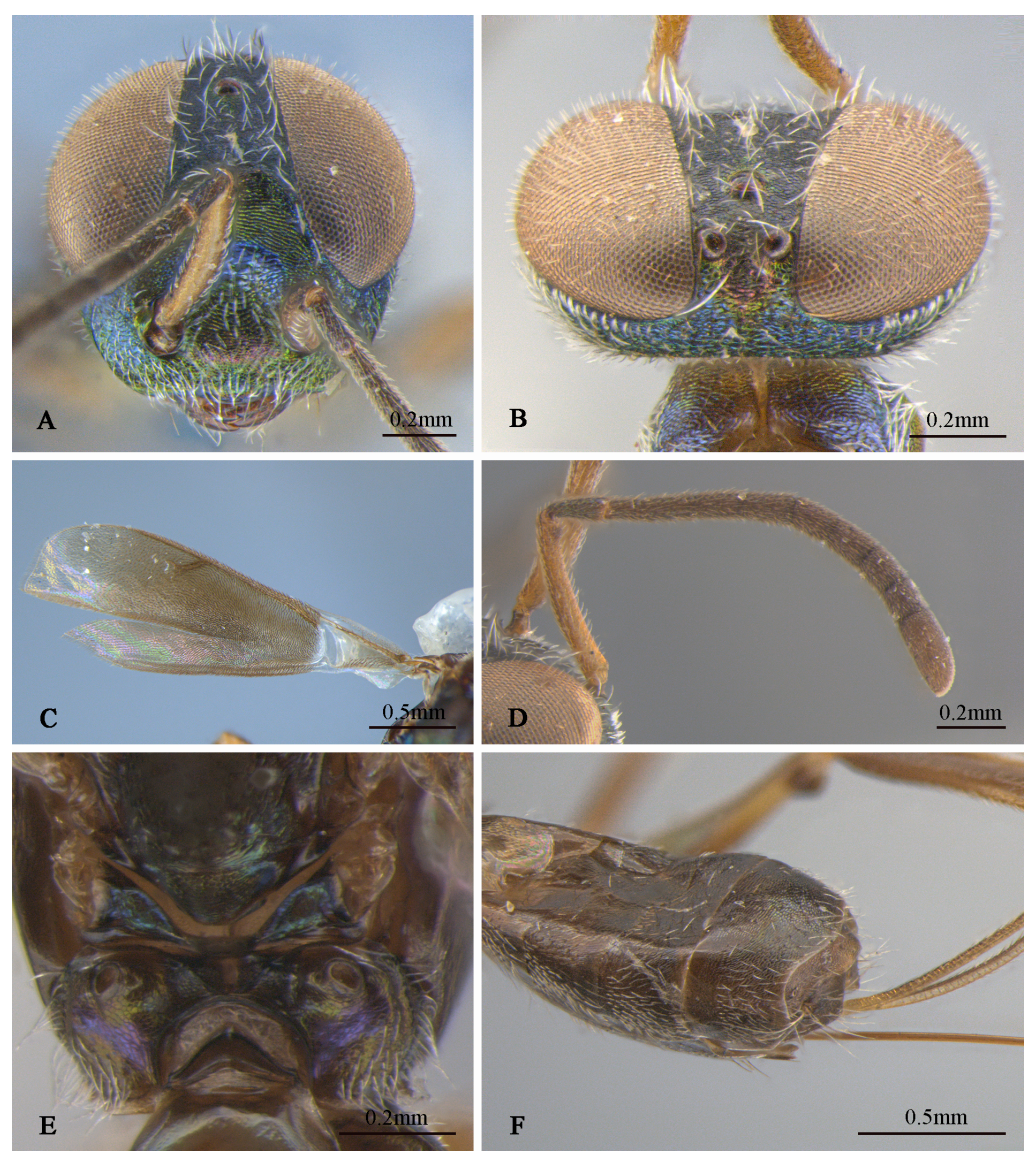
*Tineobius (Tineobius) elpisios* Li & Peng sp. nov. (holotype, FAFU-DNA962): (**A**) head, frontal; (**B**) head, dorsal; (**C**) forewing; (**D**) antenna; (**E**) propodeum; (**F**) apex of gaster, ventral.

**Type material.** Holotype: 1♀ (FAFU), Jiulianshan National Nature Reserve of Jiangxi, Jiangxi province, China|29 December 2021 to 12 February 2022|Malaise trap/DNA962. Paratypes: 1♀ (FAFU), same data as holotype|10 October 2020|Broken/DNA950; 1♀ (FAFU), Ji’nuo mount, Jinghong, Xishuangbanna, Yunnan province, China|1157 m|Flight interception trap (Black)/DNA1062.

**Etymology.** The name is derived from the Latin word for ‘*elpis*’ (‘hope’), we hope that we will never lose infinite hope, regardless of time or place.

**Description of female.** Holotype (Figure 5A,C,E and Figure 6), body length about 3.9 mm (ovipositor excluded). Head (Figure 6A,B) with green metallic luster except frons dark, vertex blue with green and coppery metallic lusters lower portion of scrobal depression with blue metallic luster, lower face coppery, dark-brown maxillary palpi. Setae of eyes and lower face white and lanceolate, setae of frons, gena and vertex white and hair-like. Vertex and temple rugose, frons mesh-like coriaceous to reticulate, scrobal depression and interantennal prominence reticulate–imbricate. In frontal view, head wider 1.1× height, distance between eyes below 0.3× distance between eyes above, distance between toruli 2.3× distance between torulus and clypeal edge, and 2.3× distance between torulus and eye. In dorsal view, head wider 1.8× length, interocular distance 0.22× head width. In lateral view, malar space 0.4× height of eye. OOL: POL: LOL: MPOD = 1.0: 4.5: 5.2: 3.8. Scrobal depression ∩-shape. Antenna (Figure 6D) dark brown in color except scape yellowish-brown, pedicel with slightly greenish-blue metallic luster; relative length (width) of scape: 93.2 (12.1), pedicel: 30.5 (10.2), 1st to 8th flagellomeres: 15.4 (10.0), 26.3 (12.0), 25.0 (12.5), 23.4 (12.9), 22.3 (14.8), 20.9 (16.4), 19.3 (17.7), 17.0 (17.9); clava 43.9 (20.0).

Pronotum (Figure 5C) and anterior region of acropleuron (Figure 5E) with yellowish-green and blue metallic lusters, posterior region of acropleuron dark, anterior convex region of mesoscutal medial lobe (Figure 5C) reddish to greenish, posterior concave region of mesoscutal medial lobe sky-blue, propodeum (Figure 5E) with bluish-purple metallic luster. Setae of pronotum, mesonotum notaulus, posterior concave region of mesoscutal medial lobe, anterior region of acropleuron and mesopectus white hair-like, posterior region of acropleuron bare. Pronotum divided medially, coriaceous to reticulate. Mesoscutum with anterior convex region of mesoscutal medial lobe punctate reticulate sculptured, lateral lobe coarsely coriaceous, acropleuron with anterior region reticulate–imbricate sculpture and posterior longitudinally alutaceous sculpture, mesoscutellar–axillar punctate–reticulate. Metanotum (Figure 6E) convex, propodeum emarginate anteromedially, plical region transverse with V-shaped plical depression, interior coriaceous sculptured. Forewing (Figure 6C) extended to near apex of gaster, basal region hyaline and partly bare, except cubital area of forewing with dense-brown hair-like setae. Setae below marginal and stigma vein brown, dense, hair-like to scalelike, beyond apex of stigma vein unobvious light short hair-like, relative length of cc: mv: pmv: stv = 3.4: 3.1: 2.2: 1.0. Front leg dark-brown; middle leg dark to brown except coxa with yellowish-green to blue metallic luster, tarsus white to dark brown, mesotibia with 9 apical pegs in 2 rows with spur brown, tarsus pale to dark, first to third joint pale, fourth and fifth brown; hind leg similar in color to middle leg, but coxa with coppery to yellowish-green metallic luster, tarsus dark to white to light brown.

Gaster (Figure 6F) brown with slightly blue and green metallic luster from different angles; Mt_6_ with posterior margin straight, posterior of Mt_7_ convex. Syntergum with posterior margin omega-like emarginated. Ovipositor sheath length about 1.6 mm, light brown to dark brown, as long as gaster, in posterior part with white band.

**Variation.** Body length is 3.6–3.9 mm, the ovipositor sheath length is in the range of 1.6–2.1 mm. cc: mv: pmv: stv = 3.4–3.7: 3.1–3.5: 2.2–2.3: 1.0, OOL: POL: LOL: MPOD= 1.0: 4.5–5.1: 5.2–5.7: 3.8–4.3. Relative length (and width) of antenna is: scape 93.2–95.8 (11.8–12.1); pedicel: 27.5–30.5 (10.2–10.5); and 1st to 8th flagellomeres: 14.9–15.4 (10.0), 26.0–26.3 (10.9–12.0), 24.4–25.0 (12.2–12.5), 22.5–23.4 (12.9–13.5), 21.1–22.3 (14.5–14.8), 19.8–20.9 (15.5–16.4), 17.5–19.3 (16.5–17.7), 15.6–17.0 (17.3–17.9); clava: 43.8–43.9 (19.8–20.0).

**Distribution.** China (Jiangsu, Yunnan).

#### 3.3.3. *Tineobius (Tineobius) longicauda* (Ferrière, 1938) n. rec. (Figure 7)

*Anastatoidea longicauda* Ferrière, 1938: 49 [9].
*Tineobius longicauda*; Bouček, 1988: 565 [4].
*Tineobius (Tineobius) longicauda*; Fusu & Ribes, 2017:13 [2].

**Material examined.** 1♀ (FAFU), Dashuikeng, Tianbaoyan National Natural Reserve, Sanming, Fujian province|21 May 2021|alt. 1070 m|Malaise trap/DNA 852.

**Figure 7:**
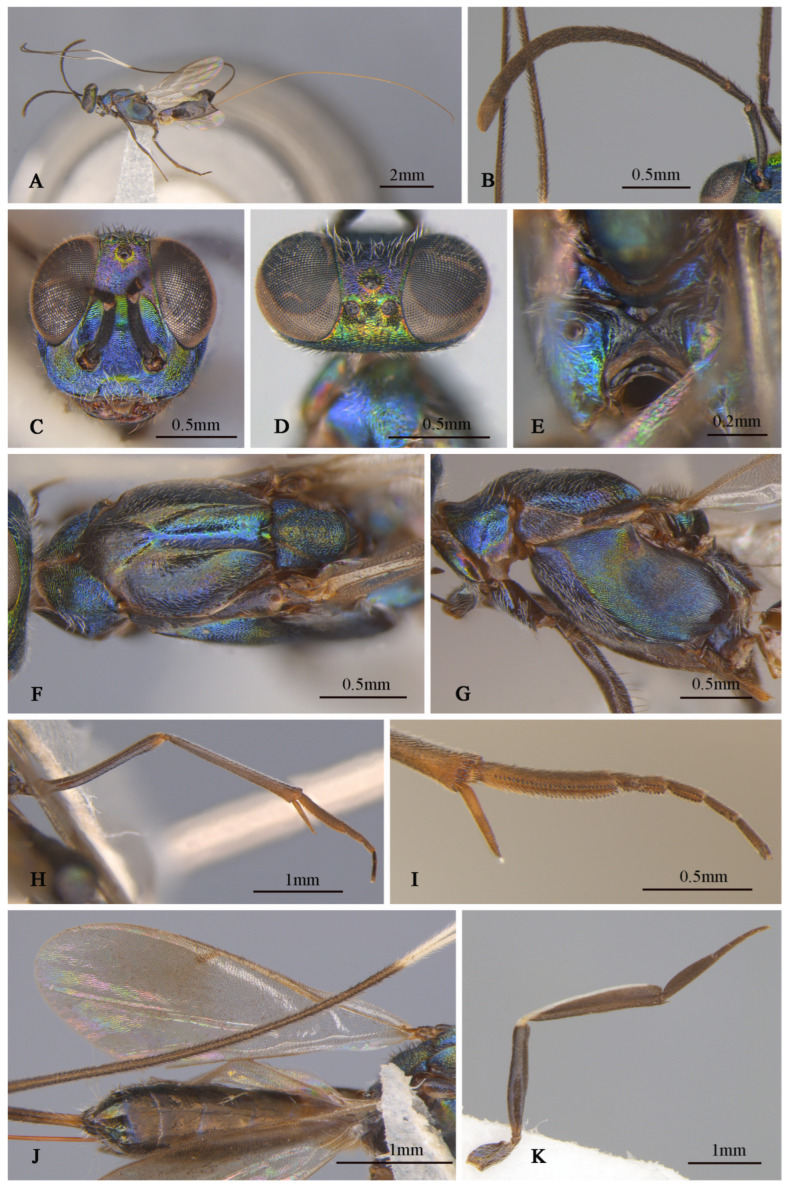
*Tineobius longicauda* (Ferrière, 1938) n. rec. (FAFU-DNA852): (**A**) body, lateral; (**B**) antenna; (**C**) head frontal; (**D**) head, dorsal; (**E**) propodeum; (**F**) mesosoma, dorsal; (**G**) mesosoma, lateral; (**H**) middle leg; (**I**) apex of mesotibia, ventral; (**J**) forewing and gaster, dorsal; (**K**) hind leg.

**Brief description of female.** Head sub-square with blue–green metal luster in front view, frons purple, lower face with hair-like setose. Antenna (Figure 7B) dark, first funicle almost same length as the pedicel, seventh funicle about twice the width of the first. Mesonotum (Figure 7F) and mesopleuron (Figure 7G) dark-blue–purple, mesoscutum posterior depressed region of mesoscutal medial lobe almost smooth. Mesoscutum (Figure 7F) with anterior convex region of mesoscutal medial-lobe coriaceous, depressed posterior region almost smooth with unobvious coriaceous, and lateral lobe coriaceous to reticulate; mesoscutum uniformly setose with white hair-like setae except depressed posterior reduced. Mesoscutellar–axillar (Figure 7F)—coarsely reticulate with distinct dark bristle-like setae. Acropleuron (Figure 7G) bare, deep blue with yellowish-green metal luster, about anterior one-quarter distinct punctate, near acropleural sulcus area imbricate, remainder alutaceous. Mesopectus more purple than acropleuron with dense, white, hair-like setae. Metanotum (Figure 7E) convex; propodeum slightly emarginate anteromedially, plical region (Figure 7E) with V-shaped, callus of propodeum deep-blue luster with white hair-like setae. Forewing (Figure 7J) with evenly setae from base to apex, behind stigma vein infuscated with little longer and more dense setae than apex area. Leg brown, lateral mesotibia with some yellow areas. Middle leg dark to brown, apex of femur lighter than others, apex with two rows of apical pegs (Figure 7I), tarsus with row of dark-brown pegs along each side of tarsomeres, last two joint more dark than other joint obviously (Figure 7H). Gaster dark, ovipositor sheath (Figure 7A,J) longer than body obviously, brown with white band.

**Distribution.** *China (Fujian), Thailand.

#### 3.3.4. *Tineobius (Tineobius) victor* Li & Peng sp. nov. (Figure 8, Figure 9, Figure 10 and Figure 11 and Figure 13)

Zoobank: urn:lsid:zoobank.org:act:6B1D2DDE-5AD5-4E1F-AA3B-DB1855A2093D.

**Figure 8:**
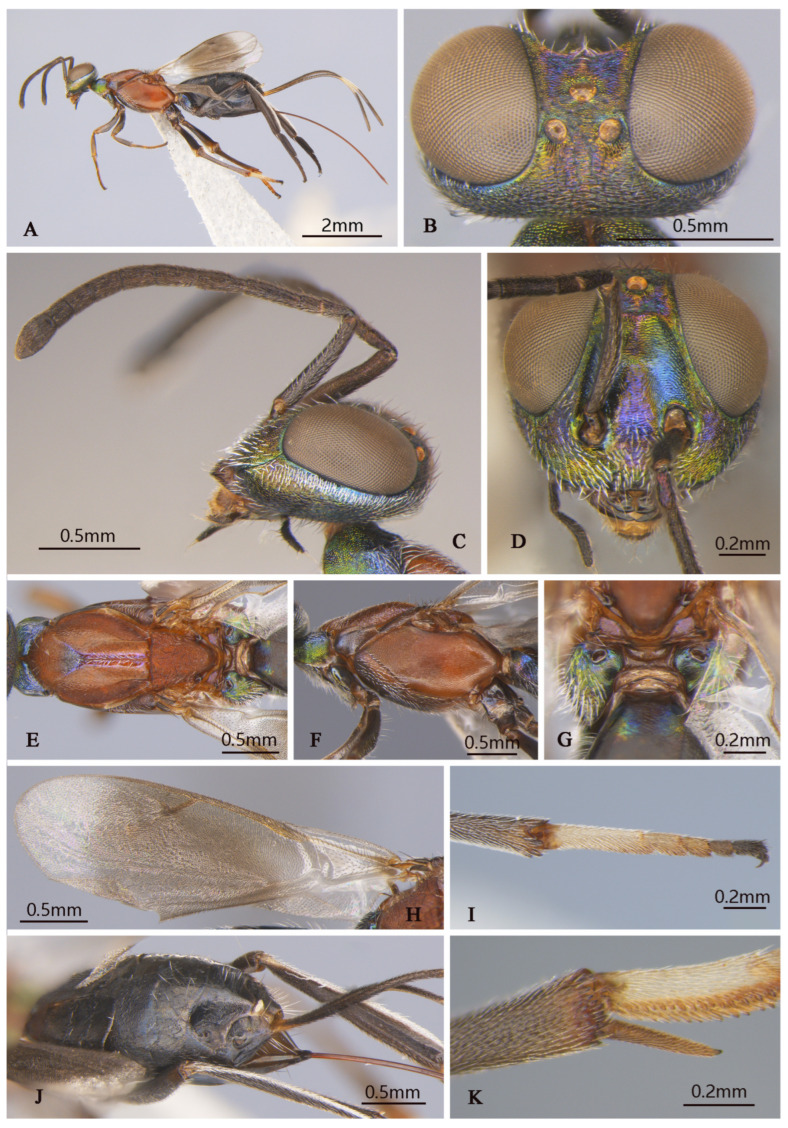
*Tineobius (Tineobius) victor* Li & Peng sp. nov. (holotype, FAFU-DNA1111): (**A**) body, lateral; (**B**) head, dorsal; (**C**) head lateral and antenna; (**D**) head, frontal; (**E**) mesosoma, dorsal; (**F**) mesosoma, lateral; (**G**) propodeum; (**H**) forewing; (**I**) apex of mesotibia, dorsal; (**J**) gaster, dorsal; (**K**) apex of mesotibia, ventral.

**Type material.** Holotype: 1♀ (FAFU), Beihang University (Shahe Campus), Beijing, China|3 September 2024|Zhenshung Huang leg./DNA1111. Paratypes: 5♀ 2♂ (FAFU), same data as holotype|7 October 2024/DNA1112, 1113, 1114, 1123, 1124 (♀), 1125, 1126 (♂); 1♀ (FAFU), Lu’er’bao sika deer breeding base, Qinhuangdao, Hebei province|15 August 2024|Mingxuan Wu leg./DNA1102; 4♀ (FAFU), Sujiazhuang village, Sweeping, Gongyi, Henan province|all ovipositor sheathes broken|26 February 2024|Weiqiong Li leg./DNA1139, 1140, 1141, 1142; 1♀ (FAFU), jujube yards, Tianjin agricultural university, Tianjin|June 2024|Weiqiong Li leg.|Malaise trap/DNA1143.

**Etymology.** The name is derived from the Latin word for “*victor*” and was chosen to commemorate the 80th anniversary of the victory of anti-fascism in World War II. It specifically honors the immense sacrifices and contributions made by the people of the world during the war.

**Description of female.** Holotype. Body length about 4.9 mm (ovipositor excluded). Head (Figure 8B–D) green with yellow metallic luster except vertex (Figure 8B) with some slightly purple metallic luster, frons with obvious deep-blue to purple metallic lusters, lower portion of scrobal depression (Figure 8D) with sky-blue metallic luster, interantennal prominence with purple metallic luster, dark-brown maxillary palpi. Setae of vertex and occiput dark, hair-like; setae of gena and lower face white and lanceolate (Figure 8C). Frons, vertex, and temple rugose; scrobal depression (Figure 8D), interantennal prominence and lower-face region reticulate. In frontal view, head wider 1.2× height, distance between eyes below 0.4× distance between eyes above, distance between toruli 1.6× distance between torulus and clypeal edge, and 1.8× distance between torulus and eye. In dorsal view, head wider 1.8× length, interocular distance 0.29× head width. In lateral view, malar space 0.4× height of eye. OOL: POL: LOL: MPOD = 1.0: 4.5: 3.9: 3.7. Scrobal depression ^-shape, dorsally delimited. Antenna (Figure 8C) dark in color, anterior region of third flagellomere with constriction; relative length (width) of scape 75.8 (13.1), pedicel 18.0 (10.3), 1st to 8th flagellomeres: 14.9 (10.0), 38.6 (9.9), 32.8 (10.8), 29.6 (11.5), 22.8 (13.5), 21.3 (13.9), 17.9 (14.1), 15.4 (13.5); clava 36.6 (19.3).

Mesosoma (Figure 8E–G) reddish to orangish brown except pronotum with green and blue metallic lusters, posterior depressed region of mesoscutal medial lobe pinkish-purple, propodeum with yellowish-green metallic luster. Setae of pronotum and anterior lobe white, hair-like, setae of posterior lobe white lanceolate; setae of lateral lobe and axillae–scutellum brown hair-like; setae of mesopectus and propodeum densely hair-like. Pronotum divided medially, reticulate sculptured. Mesoscutum (Figure 8E) with anterior convex region of mesoscutal medial lobe coriaceous, depressed posterior close to smooth, lateral lobe slightly coriaceous. Mesoscutellar–axillar complex coarsely punctate reticulate. Prepectus bare, mesh-like coriaceous. Acropleuron (Figure 8F) bare, with anterior region punctate sculpture and posterior alutaceous sculpture. Metanotum not convex, propodeum (Figure 8G) anterior margin straight, plical region transverse with ℧-shaped plical depression, interior coriaceous sculptured. Forewing (Figure 8H) extended to near apex of gaster, basal cell hyaline with sparse brown setae, below margin vein to postmarginal vein infuscated with relatively long and dense brown hair-like setae, beyond apex of postmarginal hyaline with very short and fine setae; one row of dark setae upright in submarginal vein, about 3 rows of dark setae of parastigma of submarginal vein to postmarginal vein densely, costal cell with 3 rows of short and fine dark setae, margin of costal cell with 1 row of dark setae, relative length of cc: mv: pmv: stv = 3.3: 2.2: 2.2: 1.0. Leg (Figure 8A) dark to brown except coxa with yellowish-green to bluish-purple metallic luster, hindcoxa bluish-purple obviously, mesotibia with 15 apical pegs (Figure 8K) in 2 rows and lateral with spur brown, tarsus pale to dark, first joint pale, second and third light brown, fourth and fifth dark (Figure 8I); apex of metafemur testaceous different with metatibia, metatibia and metabasitarsus compressed.

Gaster (Figure 8J) black with slightly blue metallic luster from different angles; Mt_6_ with posterior margin slightly angulate, Mt_7_ partly concealed beneath Mt_6_. Syntergum with posterior margin omega-like emarginated. Ovipositor sheath length about 3.6 mm, obviously longer than the abdomen, dark with white band, which is in posterior part of ovipositor sheath, about 1/6 of total length of ovipositor sheath.

**Figure 9:**
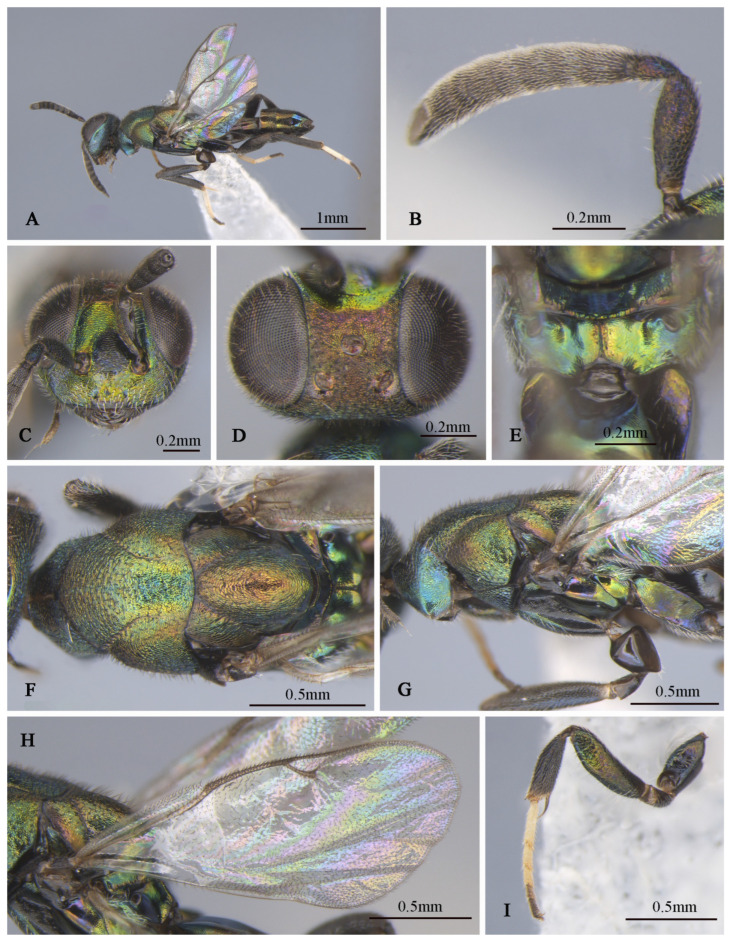
*Tineobius (Tineobius) victor* Li & Peng sp. nov. (FAFU-DNA1125): (**A**) body, lateral; (**B**) antenna; (**C**) head, frontal; (**D**) head, dorsal; (**E**) propodeum; (**F**) mesosoma, dorsal; (**G**) mesosoma, lateral; (**H**) forewing; (**I**) front leg.

**Description of male.** Body length about 3.4 mm. Head (Figure 9C,D) generally greenish-yellow with metallic luster, vertex (Figure 9D) with coppery metallic luster, scrobal depression with greenish-yellow metallic luster, interantennal prominence (Figure 9C) dark verified with other, gena region with variably greenish-blue metallic luster, maxillary and labial palpi dark brown. Setae of eyes and frons brown, hair-like, setae of vertex dark, setae of gena and lower face densely white, hair-like. Frons mesh-like reticulate to rugose, scrobal depression and interantennal prominence reticulate, lower face and gene striate-reticulate. In frontal view, head wider 1.3× height, distance between eyes below 2.0× distance between eyes above, distance between toruli 1.5× distance between torulus and clypeal edge, and 2.4× distance between torulus and eye. In dorsal view, head wider 1.7× length, interocular distance 0.4× head width. In lateral view, malar space 0.4× height of eye. OOL: POL: LOL: MPOD = 1.0: 4.1: 2.8: 2.5. Scrobal depression ∩-shape (Figure 9C). Antenna (Figure 9B) shorter than female, brown except scape and pedicel with bluish-purple metallic luster; relative length (width) of scape 42.5 (18.9), pedicel 17.5 (11.3), 1st to 8th flagellomeres: 5.7 (10.0), 19.4 (13.0), 15.5 (17.4), 15.7 (19.1), 15.7 (19.1), 15.3 (19.2), 15.8 (19.4), 15.8 (18.5); clava 32.1 (16.8).

Mesosoma (Figure 9F,G) generally with greenish-yellow metallic lusters, pronotum with greenish-blue metallic luster, mesoscutum (Figure 9F) greenish-yellow with greenish-blue metallic luster and densely short-brown setae, scutellum dark with greenish-brown metallic luster. Pronotum divided medially with densely lateral short-brown setae, mesepimeron and metapleuron with variably greenish-yellow to purplish-blue metallic luster and bare, mesepisternum dark and bare. Mesoscutum and axillae rugose, mesoscutellar coriaceous to reticulate, prepectus (Figure 9G) imbricate with greenish-yellow metallic lusters, mesepisternum, acropleuron coriaceous sculptured, mesepimeron and metapleuron coriaceous to smooth. Propodeum (Figure 9E) with variably greenish-yellow to greenish-blue metallic luster from different angles, coriaceous sculptured, densely white setae on the lateral side of the spiracle. Forewing (Figure 9H) transparent, unevenly covered brown setae, setae of basal cell sparser than other, relative length of cc: mv: pmv: stv = 3.9: 2.3: 3.2: 1.0. Front leg (Figure 9I) dark with variably greenish-yellow to purplish-blue metallic lusters, with white setae, tarsus white to dark brown, first region white, second and third pale gray, fourth and fifth dark brown, spur dark brown; middle leg similar in color to front leg, except mesotibia spur pale yellow; hind coxa similar with female in color.

Gaster (Figure 9A) dark with variably coppery or greenish-bronze metallic luster from different angles, last of gaster with blue metallic luster.

**Figure 10:**
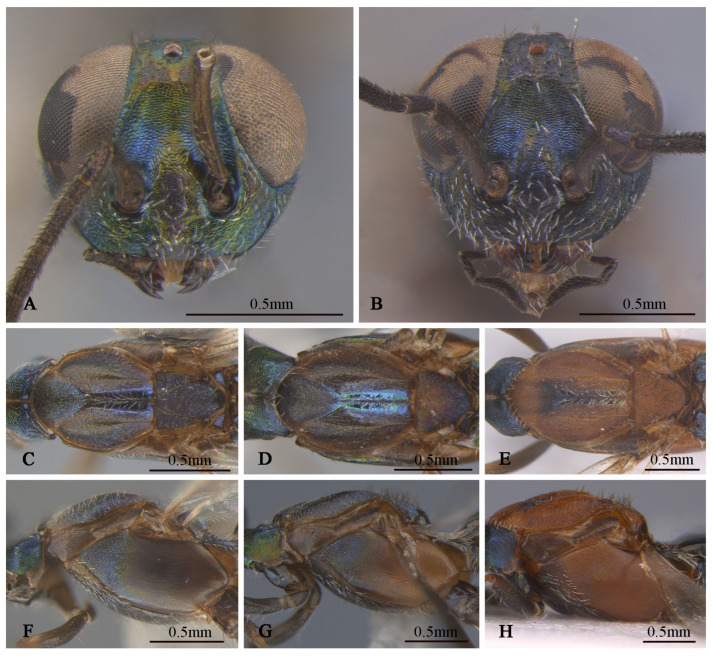
*Tineobius (Tineobius) victor* Li & Peng sp. nov variation in colour: (**A**) head, frontal; (**B**) head, frontal; (**C**) mesosoma, dorsal; (**D**) mesosoma, dorsal; (**E**) mesosoma, dorsal; (**F**) mesosoma, lateral; (**G**) mesosoma, lateral; (**H**) mesosoma, lateral ((**A**,**C**,**F**) from FAFU-DNA1142; (**D**,**G**) from FAFU-DNA1114; (**B**,**E**,**H**) from FAFU-DNA1102).

**Variation.** Female: Obvious color variations (Figure 10) are observed across the head, pronotum, mesonotum, axillae–scutellum, and propodeum, the coloration varies from bluish-grey to reddish-brown, and sometimes exhibits a green metallic luster. Certain specimens, such as DNA 1102 and 1143, differed by the lack of a distinct white band outside the ovipositor sheath. These variations are observed across the specimens, leading us to consider them as intraspecific rather than interspecific variations. The body length ranges from 3.8 to 5.3 mm, with the ovipositor sheath length ranges from 2.5 to 3.8 mm, larger individuals possess a longer ovipositor sheath. cc: mv: pmv: stv = 3.2–3.8: 1.9–2.7: 1.5–2.4: 1.0, OOL: POL: LOL: MPOD = 1.0: 3.9–4.6: 3.0–3.7: 2.3–3.7. Relative length (and width) of antenna is: scape 76.6–97.1 (13.2–17.3); pedicel 21.1–30.5 (9.8–11.9); and 1st to 8th flagellomeres: 11.1–17.9 (10.0), 30.2–43.8 (8.9–10.9), 29.6–40.2 (9.6–13.4), 27.2–34.3 (11.8–15.4), 23.5–29.0 (12.6–15.9), 21.9–26.1 (12.8–16.7), 17.1–22.3 (14.0–17.7), 13.7–22.9 (14.2–18.5); clava 27.0–46.1 (13.5–25.8). Mesotibia with 9–16 apical pegs in 2 or 3 rows.

Male: OOL: POL: LOL: MPOD = 1.0: 4.1: 2.6: 2.2; relative length (width) of scape 46.5 (17.7), pedicel 14.6 (10.5), 1st to 8th flagellomeres: 4.4 (10.0), 15.3 (12.5), 13.9 (15.3), 13.0 (18.1), 14.0 (18.2), 13.3 (17.2), 13.7 (17.2), 12.8 (16.3); clava 30.4 (15.1).

**Distribution.** China (Beijing, Hebei, Henan, Tianjing).

Observation record: China (Jiangsu).

**Figure 11:**
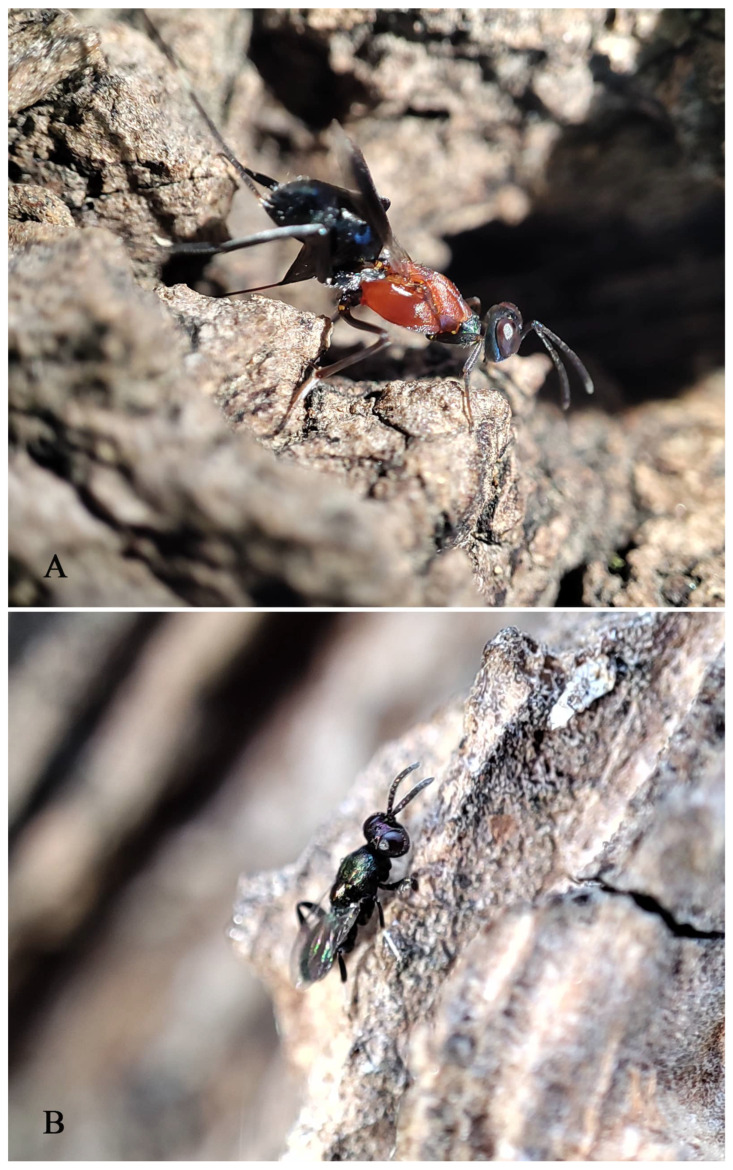
*Tineobius (Tineobius) victor* Li & Peng sp. nov.: (**A**) female; (**B**) male. Photo by Zhenshuang Huang, 7 October 2024.

**Figure 12:**
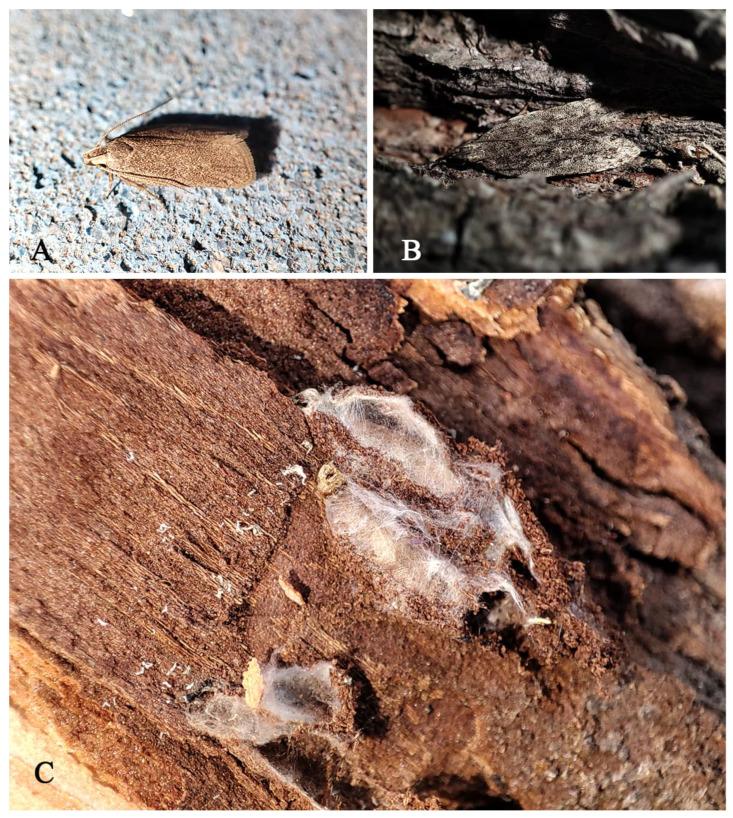
potential host of *T. victor* beneath the bark (**A**) adult of Gelechioidea gen. sp. 1; (**B**) adult of Gelechioidea gen. sp. 2; (**C**) pupa of Gelechioidea gen. sp. Photo by Zhenshuang Huang, June to September, 2024.

**Biology.** All specimens from Beijing were collected from trees of *Populus* × *canadensis*. The collector observed oviposition behavior of this species in the field and noticed the male in the same tree (Figure 11). After collecting specimens of wasps, an empty cocoon along with a suspected head capsule of a Lepidoptera insect were discovered behind the bark in the oviposition area. During the period of this wasp generation (June to October), the collector conducted long-term observations at the collection site. Based on the previous research on the host of *Tineobius* [1,2,25], we list some of the species he observed around the tree that might be related to this new species: two species of Gelechioidea (Lepidoptera) (Figure 12), *Eophyllophila* sp. and *Dolichopodomintho* sp. (Diptera: Tachinidae), and one species of Eurytomidae, one species of Braconidae, *Cheiloneurus* sp. (Hymenoptera: Encyrtidae), and *Dirhinus* sp. (Hymenoptera: Chalcididae)

**Diagnosis.** The coloration of this new species resembles that of *Tineobius tamaricis* Ribes & Fusu 2017, *Tineobius indicus* (Ferrière, 1938) and *Tineobius philippinensis* (Ferrière, 1938). The differences between this new species and *Tineobius philippinensis* and *Tineobius indicus* are shown in our key. Furthermore, it can be distinguished from *Tineobius tamaricis* by the following combination of characteristics: (1) The axillae–scutellum of *T. tamaricis* is marked with isodiametric reticulate rugose sculpture, whereas in *T. victor*, the sculpture is coarsely punctate reticulate (Figure 8E); (2) The metanotum of *T. tamaricis* is convex, while *T. victor* is not (Figure 8G); and (3) In *T. tamaricis*, the setae in the basal cell region of the forewing are pale, contrasting with the brown setae in other areas, whereas in *T. victor*, the setae on the forewings are uniformly brown (Figure 8H).

**Figure 13:**
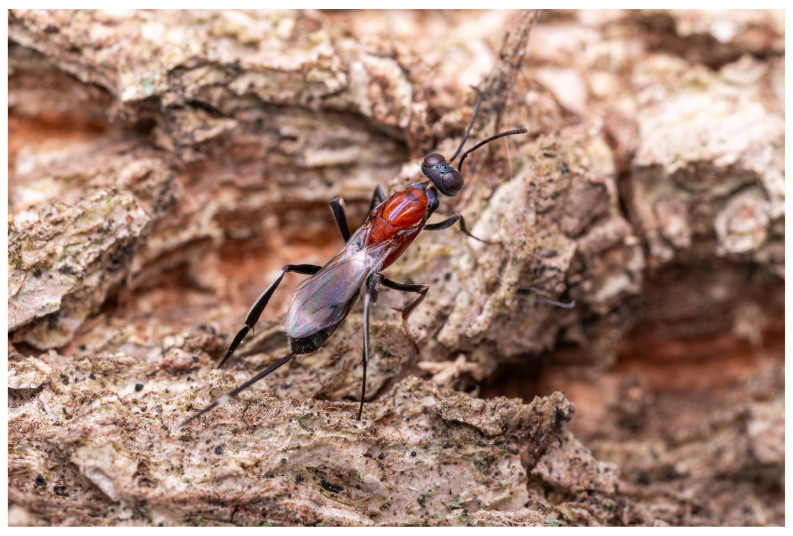
*Tineobius victor* in Nanjing, Jiangsu province. Photo by Zhenhao Feng, 3 June 2022.

**Remarks.** In addition to the existing specimen sources, we received a photograph (Figure 13) from Nanjing, Jiangsu province, which is highly likely to depict this new species. We speculate that this species has a broader distribution in China and is likely widespread across the North China Plain, north of the Yangtze River.

### 3.4. Species Excluded from Tineobius

#### *Arachnophaga (Idoleupelmus) californica* (Ashmead, 1986) comb. nov.

*Tineobius californicus* Ashmead, 1896: 14–16 [23].
*Encyrtaspis californica*; Gahan, 1943: 368–369 [26].
*Arachnophaga californica*; Noyes Universal Chalcidoidea database. Online [27].

**Remarks.** *Tineobius* was established by Ashmead, with *Tineobius citri* Ashmead, 1896 being designated as the type species. In the same year, Ashmead also described *Tineobius californicus*. However, *Tineobius californicus* was initially moved to *Encyrtaspis* Ashmead, 1904 by Gahan [26]. Subsequently, Gibson synonymized *Encyrtaspis* under *Arachnophaga* (*Idoleupelmus*), but did not update all included species to their new combinations at that time [1] (Gibson, personal communication, 2 January 2025). Consequently, Noyes included *Arachnophaga californica* in the former Universal Chalcidoidea Database on the website of the Natural History Museum of the U.K., recognizing the generic synonymy. However, this update was made without any citation. To clarify this taxonomic history, we now formally propose the correct name for this species.

### 3.5. Key to Asian Species of Tineobius Based on Females

1     Ovipositor sheaths extended for a distance equal to one-third or less of gaster; torulus near middle of face……………………………………………………………*T. (Duanellus) jacobsoni* (Ferrière)
-      Ovipositor sheaths at least as long as gaster; torulus conspicuously lower than middle of face, at or below lower orbit…………………………………………………………………………………...2
2(1) Mesonotum and mesopleuron entirely dark with metallic luster (Figure 2F,G, Figure 5C,D and Figure 7F,G)………………………………………………………………………………………………3
-      Mesonotum and mesopleuron partially or entirely reddish brown, with obscure metallic luster (Figure 8E,F and Figure 10C–H)………………………………………………………………………..5
3(2) Ovipositor sheaths almost four-times longer than gaster (Figure 7A), forewing with evenly short hair-like setae, behind marginal veins with relatively sparse, short, hair-like setae and without infuscated region (Figure 7J)…………………………………………………………………………………………………………………………………………….*T. (Tineobius) longicauda* (Ferrière)
-      Ovipositor sheaths almost equal in length to gaster (Figure 2J and Figure 5A); basal cell of forewing bare or mostly bare; behind marginal veins with infuscated region and relatively dense scalelike to hair-like setae (Figure 2H and Figure 6C)………………………………………………………………………………………………………………………………………………………………...4
4(3) Ovipositor entirely dark (Figure 2J); lower face with scalelike setae (Figure 2D); posterior depression region of mesoscutum without distinct white, hair-like setae (Figure 2G); whole basal region of forewing almost hairless (Figure 2H)………………………………………………………………………………………………………………………………...*T. (Tineobius) brachartonae* (Gahan)
-      Ovipositor dark with white band (Figure 5A); lower face with lanceolate setae (Figure 6A); anterior region of mesonotum with distinct white hair-like setae extending to posterior depression region (Figure 5C); cubital area of forewing with dense-brown hair-like setae (Figure 6C)………………………………………………………………………..*T. (Tineobius) elpisios* Li & Peng sp. nov.
5(2) Gt_1_ and Gt_2_ pale to yellowish-brown………………………………………………………………………………………………………………………………………………...*T. (Tineobius) indicus* (Ferrière)
-      Gaster entirely black (Figure 8J)…………………………………………………………………………………………………………………………………………………………………………………………5
6(5) Pronotum dark metallic; forewing with lanceolate to scalelike setae below parastigma and marginal vein, basal cell bare……………………………………...*T. (Tineobius) philippinensis* (Ferrière)
-      Pronotum typically exhibiting green or dark-blue metallic luster (Figure 8E); forewing with hair-like setae from base to apex, no scalelike setae (Figure 8H)……………………………………………………………………………………………………………………………………………………………………………….*T. (Tineobius) victor* Li & Peng sp. nov.

## 4. Discussion

In this research, we report the second known species of *Tineobius* within the Palaearctic region. The initial species, *Tineobius tamaricis* Ribes & Fusu, found in Spain, is suspected to be an introduced species rather than a native endemic. This assumption is supported by the fact that no males were reared over a four-year period, suggesting that it may be a thelytokous species [2]. If this is the case, it would enhance the probability of the species having been introduced from another region [28]. In contrast, our newly discovered species includes male specimens. Therefore, we suggest that *Tineobius* might be more widely distributed than previously recognized.

Our collection currently has three additional *Tineobius* species from Fujian and Guangdong provinces that have yet to be described, with each species represented by a single specimen. Although comprehensive morphological descriptions are pending, we have uploaded two COI sequences to facilitate future identification. Continued long-term sampling and research will be necessary to properly characterize these taxa.

**Figure 14:**
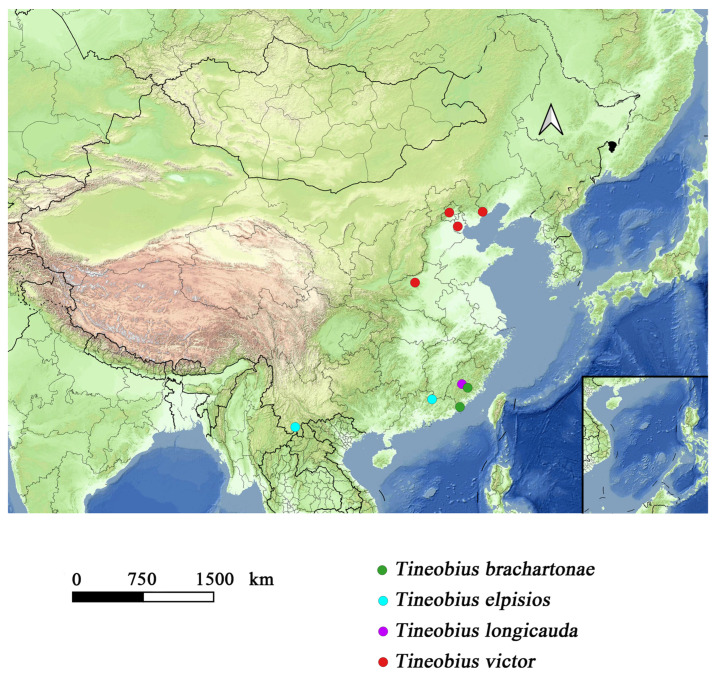
Distribution map of the recorded *Tineobius* in China.

**Figure 15:**
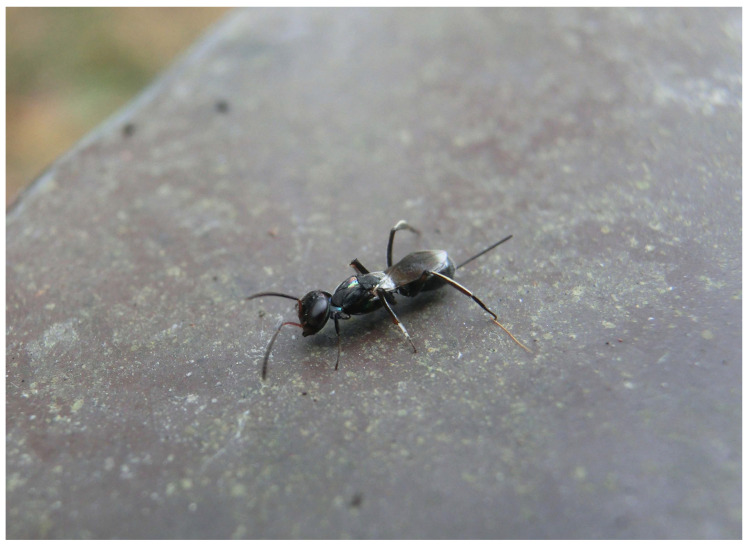
*Tineobius brachartonae* in Zhanghua, Taiwan province. Photo by Changlang Liu, 1 January 2023.

**Figure 16:**
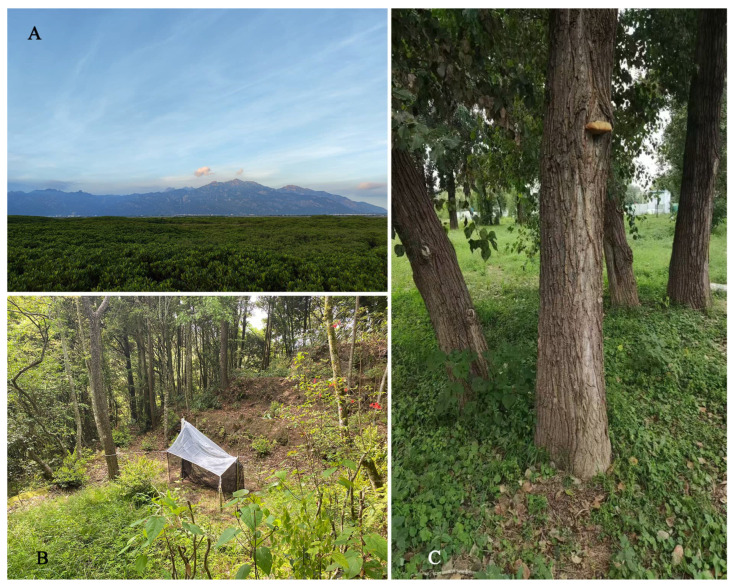
Habitat of *Tineobius* from China: (**A**) *Tineobius brachartonae*, mangrove forest; (**B**) *Tineobius longicauda*, subtropical evergreen broad-leaved forest; (**C**) *Tineobius victor* sp. nov., campus lawn/temperate deciduous forest.

A distribution map of *Tineobius* was constructed based on our specimens (Figure 14), which highlights the considerable potential for undiscovered diversity. According to the data from the iNaturalist platform, at least four species of *Tineobius* have been observed in Taiwan province, one of which can be confidently identified as *Tineobius brachartonae* (Figure 15). Not only in China (Figure 16), but we also hope that local taxonomists will discover and describe more species of *Tineobius*.

## Figures and Tables

**Table 1:** Sequenced taxa and GenBank accession numbers.

Code	Species	Sex	Genbank Accession No.
FAFU 481 FAFU 1081 FAFU 1098 FAFU 1103 FAFU 1104 FAFU 1108 FAFU 1109 FAFU 962 FAFU 1062 FAFU 852 FAFU 1076	*Tineobius brachartonae* *Tineobius brachartonae* *Tineobius brachartonae* *Tineobius brachartonae* *Tineobius brachartonae* *Tineobius brachartonae* *Tineobius brachartonae* *Tineobius elpisios* sp. nov. *Tineobius elpisios* sp. nov. *Tineobius longicauda* *Tineobius* sp. 1	female female female female male female female female female female female	PV500981 PQ664482 PQ664483 PQ664484 PQ664485 PQ664486 PQ664487 PV500983 PV500984 PV500982 PV500985
FAFU 1101 FAFU 1102 FAFU 1111 FAFU 1114 FAFU 1124 FAFU 1125 FAFU 1126	*Tineobius* sp. 2 *Tineobius victor* sp. nov. *Tineobius victor* sp. nov. *Tineobius victor* sp. nov. *Tineobius victor* sp. nov. *Tineobius victor* sp. nov. *Tineobius victor* sp. nov.	female female female female female male male	PV500986 PV500987 PV500988 PV500989 PV500990 PV500991 PV500992
FAFU 1139	*Tineobius victor* sp. nov.	female	PV500979
FAFU 1140	*Tineobius victor* sp. nov.	female	PV500980
FAFU 1141 FAFU 1142 FAFU 1143	*Tineobius victor* sp. nov. *Tineobius victor* sp. nov. *Tineobius victor* sp. nov.	female female female	PV500993 PV500994 PV500995

## Data Availability

The original contributions presented in this study are included in the article/Supplementary Material. Further inquiries can be directed to the corresponding author.

## Supplementary Materials

The following supporting information can be downloaded at: https://www.mdpi.com/article/10.3390/insects16060597/s1, Table S1: Genetic distance of COI within species under K2P model; Table S2: Interspecific pairwise distance of Tineobius based on COI sequences (%).
