# Supplementary material for: Beneath the Bark and Beyond the Known: The First Record of Tineobius Ashmead (Hymenoptera: Chalcidoidea: Eupelmidae) in China with a Description of Two New Species
Source: Insects. 2025 Jun 5;16(6):597. doi: 10.3390/insects16060597 (PMC12193237; doi:10.3390/insects16060597)
Supplement: Supplementary file 1 [file insects-16-00597-s001.zip › Table S1 Genetic distance of COI within species under K2P model.pdf]

Beneath the Bark and Beyond the Known: The First Record of *Tineobius* Ashmead (Hymenoptera: Chalcidoidea: Eupelmidae) in China with a Description of Two New Species

Zixuan Li [1,2,3], Haoran Liao [1,2,3], Shirui Xu [1,2,3], Haitian Song [4] and Lingfei Peng [1,2,3]*

[1] Biological Control Research Institute, Fujian Agriculture and Forestry University, Fuzhou 350002, China; lizixuan1813@163.com (Z.L.); liaohaoran76@gmail.com (H.L.); xushirui10050@163.com (S.X.)
[2] China Fruit Fly Research and Control Center of FAO/IAEA, Fuzhou 350002, China
[3] State Key Laboratory of Ecological Pest Control for Fujian and Taiwan Crops, Fuzhou 350002, China
[4] Fujian Academy of Forestry, Fuzhou 350012, China; haitiansong@126.com
* Correspondence: lingfeipeng@fafu.edu.cn

Supplementary Material

Table S1. Genetic distance of *COI* within species under K2P model

Species	Distance (%)
Tineobius brachartonae	0-0.003
Tineobius elpisios	0.031
Tineobius longicauda	NA
Tineobius sp.1	NA
Tineobius sp.2	NA
Tineobius victor sp.nov.	0-0.032
