# Supplementary material for: Beneath the Bark and Beyond the Known: The First Record of Tineobius Ashmead (Hymenoptera: Chalcidoidea: Eupelmidae) in China with a Description of Two New Species
Source: Insects. 2025 Jun 5;16(6):597. doi: 10.3390/insects16060597 (PMC12193237; doi:10.3390/insects16060597)
Supplement: Supplementary file 1 [file insects-16-00597-s001.zip › Table S2 Interspecific pairwise distance of Tineobius based on COI sequences (%).pdf]

Beneath the Bark and Beyond the Known: The First Record of *Tineobius* Ashmead (Hymenoptera: Chalcidoidea: Eupelmidae) in China with a Description of Two New Species

Zixuan Li [1,2,3], Haoran Liao [1,2,3], Shirui Xu [1,2,3], Haitian Song [4] and Lingfei Peng [1,2,3]*

[1] Biological Control Research Institute, Fujian Agriculture and Forestry University, Fuzhou 350002, China; lizixuan1813@163.com (Z.L.); liaohaoran76@gmail.com (H.L.); xushirui10050@163.com (S.X.)
[2] China Fruit Fly Research and Control Center of FAO/IAEA, Fuzhou 350002, China
[3] State Key Laboratory of Ecological Pest Control for Fujian and Taiwan Crops, Fuzhou 350002, China
[4] Fujian Academy of Forestry, Fuzhou 350012, China; haitiansong@126.com
* Correspondence: lingfeipeng@fafu.edu.cn

Supplementary Material

Table S2. Interspecific pairwise distance of *Tineobius* based on *COI* sequences (%)

	Species	1	2	3	4	5	6	7
1	*Tineobius brachartonae*		12.3-13.0	20.1-20.3	15.8-16.0	19.1-19.5	15.5-17.2	16.6-16.8
2	*Tineobius elpisios*			19.3-20.9	14.3-15.5	17.2-17.5	13.8-16.0	16.6-17.2
3	*Tineobius longicauda*				21.9	24.2	22.2-24.2	22.2
4	*Tineobius* sp.1					14.5	17.9-19.9	20.7
5	*Tineobius* sp.2						20.1-21.9	23.4
6	*Tineobius victor* sp.nov.							17.6-19.9
7	*Tineobius tamaricis*							

1
